# Some Points in the Diagnosis and Treatment of Disease of the Aortic Valves

**Published:** 1909-04-10

**Authors:** S. Russell Wells

**Affiliations:** Physician to the Seamen's Hospital, Greenwich; and Assistant Physician and Pathologist to the National Hospital for Diseases of the Heart, Soho Square


### Hospital Clinics.

### SOME POINTS IN THE DIAGNOSIS AND TREATMENT OF DISEASE OF THE AORTIC VALVES.

By S. RUSSELL WELLS, M.D., B.Sc., M.R.C.P.; Physician to the Seamen's Hospital, Greenwich; and Assistant Physician and Pathologist to the National Hospital for Diseases of the Heart, Soho Square.

(A Lecture delivered at the Polyclinic.)

I propose this evening to discuss disease of the aortic valves. I have recently been going through the notes of the last 500 completed cases I have seen at the Hospital for Diseases of the Heart, and certain facts in connection with the cases of aortic trouble have forcibly impressed themselves on my mind. The classical text-book description of a typical case of aortic regurgitation is familiar to you all, but for the sake of clearness you will, no doubt pardon me if I cite a case and recapitulate the principal symptoms, signs, and anatomical and physiological pathology.

### Two Cases of Aortic Regurgitation.

Henry W., general labourer, aged 46, came to hospital on September 26, 1907, complaining of pains in the chest and pain running down the left arm; at times he "felt a fluttering" in his chest (obviously palpitation), and occasionally had dizzy faint feelings. These symptoms had been present more or less for four years, but without shortness of breath or marked dyspepsia. Recently he had suffered a good deal from sleeplessness, and he explained that "the fluttering" in his chest was what kept him awake. There was nothing of importance in his family history, and the only previous illnesses that he knew of were childish ailments such as measles, whooping-cough, and two attacks of influenza. There was no history of rheumatic fever, scarlet fever, chorea or syphilis. He had drunk somewhat freely and his work had always been heavy and laborious. On examination he was found to be a well-developed, rather stout man; the pulse was of a typical collapsing or water-hammer character, the carotid, brachial and other arteries showed marked and visible pulsation. The apex beat was situated in the sixth interspace, a little outside the left nipple line, and was sudden and forcible. On percussion the area of cardiac dulness was not found to be enlarged to the right, but was considerably increased downwards and to the left. On auscultation, a well-marked diastolic murmur was heard with maximum intensity at the sternal end of the second right costal cartilage, but it could also be heard down the right side of the sternum. A second sound was audible on listening over the arteries in the neck.

On everting the lower lip and placing a microscope slide on the mucous membrane, the mucous surface was seen to blanch and redden with the pulse. I may here mention that I have found this a much more convenient method of demonstrating a capillary pulse than rubbing the forehead or pressing the nail. In short, the case was as clear a one of pure aortic regurgitation as anyone could wish to see. The man's age, and absence of any history of rheumatism or sudden symptoms made it almost certain that the regurgitation was due to atheromatous changes in the aortic valves. Now, what history are we to give to the morbid conditions? and what is the explanation of the symptoms?

As the result of his strenuous occupation, aided no doubt by the alcohol he indulged in, probably in the first instance his blood-pressure rose and increased strain was put upon the aortic valves, in consequence of which atheromatous changes occurred: thickened, and shrunk, or puckered, they no longer perfectly closed the aortic orifice during the ventricular diastole, and consequently regurgitation took place into the left ventricle. As this regurgitation took place while the muscle was at rest, and blood was also flowing from the left auricle, the ventricle would tend to become overcharged and distended; consequently at the next systole it would have more work to do, and more blood to discharge into the aorta.

As a result the left ventricle became dilated, but this put the heart muscle at a disadvantage, for by a physical law, which I need not pause to discuss here, the exertion of each muscular fibre, if the pressure of the blood in the aorta is to remain constant, varies as the cube of the radius of curvature of the ventricle. You see therefore that if there was no hypertrophy, and the diameter of the ventricle was doubled, the work of the muscle would be increased eight-fold. In this case, as the nutrition of the ventricle was good, increased work meant increase of muscular fibre, and hypertrophy of ventricle occurred; thus the dilatation and hypertrophy compensated for the regurgitation.

Since, however, the left auricle had to discharge its blood into a ventricle which was being partially filled by blood under pressure from the aorta, it also would become somewhat enlarged and hypertrophied. It is, therefore, easy to account for the increased size of the left side of the heart as shewn by percussion, and since at this stage no increased work is thrown upon the right side we can easily understand why the increase of cardiac dulness was entirely downward and to the left, and not to the right as we find it in mitral disease.

The diastolic murmur was caused by the blood whistling under pressure from the aorta, through the chink left by the imperfectly closed aortic valves into the dilating or passive left ventricle. The reason that it was best heard at the second costal cartilage was that the sound was conducted along the wall of the aorta which here comes nearest to the surface. It may be asked why it was not heard at the apex of the heart, since one imagines that the regurgitated stream flowing into the ventricle would impinge upon the apex, and that the sound would be carried in the direction of the flow of the blood; but we must remember that the amount of blood regurgitated is necessarily much less than that flowing in from the auricle, and as the two streams meet the aortic current will be deflected. It is true in certain cases that an aortic diastolic murmur may be heard with maximum intensity about the second interspace, and be conducted up and down the sternum, lost as we move the end of the stethoscope to the left, and heard again over the apex beat; but I should say more frequently it is inaudible at the apex. What is the explanation of the water-hammer pulse? If we take a sphygmographic tracing of such a pulse, we find that there is a sudden and greater rise of pressure than usual. This is due to the enlarged and hypertrophied ventricle sending a larger quantity of blood more forcibly than normally into the arteries, which, during diastole, had probably contained blood at somewhat less than normal pressure. This sudden rise is followed by an equally sudden and rapid fall, due to the fact that the blood is not held up by the aortic valves, but some of it regurgitates into the ventricle. As a result of the sudden distension of the arteries, their elastic recoil is greater than normal, and consequently the fall is followed by a well marked dicrotic wave, and so during the diastole the pressure is below normal.

Anything which increases the strain on the aortic valves, anything which aids the fall of blood, will increase the water-hammer character of the pulse; consequently it is brought out more markedly by lifting the arm. No doubt the faintness and dizziness complained of in well marked aortic incompetence on suddenly rising from a stooping posture and the like, is due to the action of gravity causing diminished pressure in the cerebral vessels.

Palpitation is no doubt accountable for by the enlarged and hypertrophied ventricle beating more violently, and by nutritional changes at times increasing the rate of the heart's beat. But how are we to account for the pain down the left arm? On looking over the notes of a large number of cases of valvular lesion I have been struck with how uncommon this symptom is in mitral disease. In cases of aortic disease I should say that it is distinctly most frequent in those due to atheroma. I should not like to insist too strongly on this point, but my impression is that it is distinctly in favour of atheromatous changes in the valves and in the neighbouring part of the aorta. If this is so why should the pain radiate down the arm instead of being confined to the chest? We must first remember that any pain is not really felt by the part affected, but by the brain. When we say we feel a pain in our hand, what we really mean is that a certain sensation has reached our brain by the sensory nerves coming from the hand, and that by experience we have learnt to associate this impression with the hand. The instance of the man whose arm had been amputated feeling a tingling in his fingers is known to you all. A study of development shows that the body may be mapped out into zones, each of which corresponds to the area of distribution of the posterior root of one of the spinal nerves. The first dorsal root conveys impressions from the ulnar side of the forearm and lower half of the upper arm, while the second dorsal is concerned in the sensation of the upper half of the inner side of the upper arm, the third and fourth cervical, correspond to the shoulder and a portion of the neck. But the sympathetic nerves also correspond to segments of the cord, and the sympathetic nerves supplying the aorta from the valves to the level of the origin of the innominate artery may be considered to correspond to the third and fourth cervical and first and second dorsal segments. Impulses arriving by the sympathetic fibres from the aorta, reaching the grey matter of the third and fourth cervical and first and second dorsal segments, may radiate to the posterior horn, and consequently these impressions be referred to the area of distribution of the corresponding sensory fibres. Sometimes, but far less frequently, pain is complained of in both arms.

I have taken the case just cited because it was so clear and simple, but in my experience it is comparatively rare to find a simple aortic diastolic murmur with accurate location. Far more commonly the murmur is double, and we find a systolic as well as a diastolic murmur. Let me quote another case.

Elizabeth K., aged 48, caretaker, complained of palpitation, feeling of tiredness, and pain in the left side of chest for two months. Sleep poor; slight dyspnœa on exertion; a faint diastolic and louder systolic murmurs heard with maximum intensity in the right second interspace, near the sternum, and conducted down and across the sternum; the systolic, but not the diastolic, heard at the apex beat, which was situated in the sixth interspace an inch outside the nipple line. No second sound was audible in the vessels of neck; the area of cardiac dulness was long, enlarged to the left, but not to the right of the sternum; the apex beat was forcible, but diffuse; both water-hammer and capillary pulse were present; a heaving pulse could be detected in the veins, and there was a well-marked Durozier's sign. As I have not mentioned Durozier's sign, I may explain that it is a double murmur heard on listening with the stethoscope rather firmly pressed on one of the great arteries, the femoral for choice; it is practically diagnostic of aortic regurgitation, and is produced as follows: The pressure of the stethoscope causes a constriction of the artery. The blood rushing through this narrow portion into the wider part beyond at the systole of the pulse produces a systolic murmur, which can be heard in a healthy subject. When marked aortic regurgitation is present there is a backward flow during the diastolic phase, and this causes the diastolic murmur.

What is the explanation of this case? Had the woman in question aortic stenosis as well as regurgitation? I think not. The marked water-hammer pulse, the capillary pulse, and Durozier's sign are against it, as is the absence of thrill. While a double aortic murmur may be present without stenosis, so also a presystolic murmur audible at the apex, is not certain proof of mitral stenosis. I will not weary you by repeating the full account of such a case, but will content myself by assuring you that I have heard such a murmur in several cases. How are we to account for this murmur, which was first described by Austin Flint, and is called after him? Let us think for a minute of what is happening in the left ventricle; the blood regurgitating from the aorta into the ventricle impinges on the anterior or aortic flap of the mitral valve, and so narrows the orifice through which the blood flowing from the auricle has to pass: thus with the auricular systole a presystolic murmur is produced, though there is no organic mitral stenosis.

### A Case of Aortic Stenosis.

I now want to direct your attention to a case of a very different character. John S., aged 54, hotel porter, was admitted to hospital on September 25, 1907. He had rheumatic fever at the age of 25, and suffered on and off for 25 years from palpitation; but this had never been very bad until a year before admission, when he had severe dyspnœa as well, and the palpitation became more continuous. On examination, the apex beat was found in the sixth interspace, ¾ inch external to the left nipple line; the impulse was heaving and forcible; the area of cardiac dulness extended ¾ inch beyond the left nipple line, though very little increase was detected to the right; the pulse was weak, small, and slightly irregular, rate 70. Auscultation showed a long, blowing, systolic murmur all over the præcordia; it was difficult to say what was the point of maximum intensity, but a systolic thrill could be felt over the base of the heart. A very short, soft, faint diastolic murmur was occasionally audible in the aortic area and along the right border of the sternum. The pulmonary second sound was accentuated, and in the mitral area the second sound was reduplicated. There was some dulness at the base of both lungs; the liver was palpable and tender three inches below the costal margin; there was no œdema of the legs, nor albuminuria.

For the first fortnight he seemed to improve, had no bad symptoms, and complained of no pain. The diastolic murmurs varied considerably: some days more or less distinct, on others inaudible, or very indefinite and heard only over a very limited area. On October 13 he began to be troubled with dyspnœa, and the pulse became extremely irregular and at times very weak; there was slight increase of cardiac dulness to the right of the sternum and some œdema of the lungs. Albuminuria appeared, but apart from the discomfort of the dyspnœa he had no pain. He gradually became weaker, and died on Oct. 27.

*Post-mortem*, the heart weighed 21 ounces. There was great concentric hypertrophy of the left ventricle with some dilatation. The right side was moderately dilated, the muscle was pale and showed pheasant-wing markings, the pulmonary and tricuspid valves were healthy, but the tricuspid orifice was dilated and the valves incompetent to the water-test. The mitral valves showed slight thickening at the edges, but no vegetations. The orifice admitted four fingers readily and showed slight incompetence to the water-test. The aortic valves showed extreme change; there was a large deposit of calcareous matter; two of the valves were fused into one calcareous mass, making the orifice narrow and slit-like. It is probable there was little or no regurgitation, because at the edge of the thickened and hardened valves there was a thin, narrow band of flexible membrane, which closed the chink when water was poured into the aorta.

We have here a case of aortic stenosis of long standing, in which muscular degeneration led to mitral regurgitation, increased pulmonary pressure, and tricuspid regurgitation. Let us trace out in detail the probable course of events. As the result of his rheumatic fever the aortic valves became thickened, and, though still competent, the orifice was narrowed. The left ventricle had increased difficulty in driving the blood during the systole through the narrowed aortic orifice. The ventricle became hypertrophied and the systole was prolonged, increased strain being thrown upon the mitral valve as a result of this and the comparatively slow delivery of blood into the aorta. The pulse wave would rise slowly, and would not reach the same height as normal, because much of the force of the hypertrophied ventricle would be used up in overcoming the resistance at the aortic orifice. Since the systole was prolonged while the ventricle was driving its steady stream of blood through the narrowed orifice, the systolic pulse-wave would be long sustained and fall slowly. Sometimes the ventricle makes as it were two attempts to get the blood past the obstruction, and we find on a sphygmographic tracing that the top of the pulse curve is notched, the so-called pulsus bisferiens. We see that the pulse tracing of aortic stenosis is exactly the converse of that of aortic regurgitation, and to the finger the pulse feels small, weak, and prolonged, and utterly different to the collapsing water-hammer pulse.

### Stenosis with Regurgitation.

Having discussed cases of pure aortic regurgitation and one of practically pure aortic stenosis, which is very much rarer, let us now consider cases where there is both aortic stenosis and regurgitation. I must first observe that aortic stenosis with regurgitation is frequently diagnosed by the inexperienced in cases where there is regurgitation alone, the observer being misled by such a case as the second I mentioned, where a double murmur is audible over the aortic area. On June 27, 1907, I saw at the Heart Hospital, Samuel B., aged 33, a fitter's labourer. There was nothing of importance in his family history. The patient had had three attacks of rheumatic fever, which had occurred when he was seven, 16, and 25. He complained of pain at the heart, palpitation, and occasional dizziness. On examination he was found to have a large heart of the shape typical of both aortic regurgitation and stenosis. You will have remarked that so far as percussion is concerned, both lesions give the same indication. The apex beat was diffuse and forcible, a well marked double murmur was audible over the aortic area and down the sternum; the diastolic portion could be heard at the apex, and the systolic was audible in the vessels in the neck. On placing the hand on the base of the heart a well marked thrill could be felt, and a thrill was also palpable in the carotids. The pulse was neither large, sudden, nor collapsing, nor was it of the small prolonged character typical of aortic stenosis. There was no capillary pulse, nor could Durozier's sign be made out. I diagnosed the case as one of combined stenosis and regurgitation of the aortic valves, in which the stenosis was well marked. The main points to bear in mind are these. The diagnosis depends chiefly upon the character of the pulse; if with a double murmur you have a well marked water-hammer pulse, capillary pulse, Durozier's sign, and pulsating carotids, you may pretty safely exclude stenosis; but if the murmurs point to the combined lesion, and percussion and palpation confirm it, while the pulse is slow, small, and though it may be slightly collapsing, is far from being a typical water-hammer, then some stenosis is probably present. The character of the impulse of the heart is of some importance: in stenosis hypertrophy is the first and main change in the ventricle; while in regurgitation dilatation is more marked. Consequently the heart in stenosis tends to be smaller and the impulse slower and more heaving than in regurgitation; the apex beat in stenosis sometimes seems to push against or lift the chest wall, whereas in regurgitation it seems to strike it.

### Regurgitation without Valvular Lesions.

So far I have dealt with cases in which the stenosis or regurgitation has been caused by changes in the valves themselves. Is aortic regurgitation possible without the actual valves being affected? The following is a case in point:

Roland S., aged 33, cabdriver, was admitted to the hospital, November 18, 1907. As he was not quite clear-headed on admission, the history had to be obtained from friends, and was probably imperfect. The only previous known illnesses were inflammation of the lungs and four attacks of influenza. For several months he had complained of pain over the cardiac area and some dyspnœa; for the last few weeks the dyspnœa had been worse, and he had suffered from insomnia; he had had a cough for about a week. On examination he was found to be a well nourished man, the capillaries of the face were dilated, the arteries hard and thickened, the pulse was peculiar, the wave rising rapidly and falling quickly, but there was considerable tension between the beats; in fact, it may be permissible to describe it as a water-hammer pulse with a very high tension.

The apex beat was in the sixth interspace, two inches external to the nipple line, the left limit of cardiac dulness was two inches external to the nipple line, and the right a little over an inch to the right of the edge of the sternum. On listening to the heart, a loud blowing systolic murmur was heard, having two points of maximum intensity —namely over the aortic area and at the apex; it was difficult to say at which of these two it was the louder. A soft blowing diastolic murmur was heard with maximum intensity at the aortic area. The liver extended three inches below the costal margin and was tender, the lungs were dull at both bases, but moist crepitations could be heard. The urine was scanty, high-coloured, and contained albumin, there was slight œdema of the legs. On November 21 he became delirious, and died eight hours later.

The aorta was found tremendously dilated and atheromatous about the valves, but the valves themselves were absolutely healthy. The aortic orifice admitted three fingers easily, and it was obvious that aortic regurgitation had taken place during life. The left ventricle was greatly hypertrophied and dilated. The muscle was pale and showing signs of fatty degeneration, the mitral valves were healthy, but the mitral ring was dilated. The tricuspid and pulmonary valves were all healthy. Both auricles were enlarged, and fluid was present in both pleural cavities. Organised lymph covered the lower lobe of the right lung, which was carnified. Both kidneys showed typical changes of contracting large white kidney. The liver was enlarged and nutmeg. Here we had to do with a case of renal disease; as a result of the increased peripheral resistance which this disease produces the blood-pressure rose and caused hypertrophy of the heart. While the hypertrophy could keep pace with the increasing peripheral resistance all went well, so far as the heart was concerned; but the high blood-pressure caused atheroma and dilatation of the aorta; this, assisted, no doubt, by the hypertrophy and dilatation of the left ventricle, caused the aortic orifice so to enlarge that the normal aortic valves became incompetent to close it, and the symptoms and signs of aortic regurgitation occurred, followed by those of mitral regurgitation.

### Death in Aortic Regurgitation.

Now let us discuss the ways in which aortic regurgitation causes death. There are two main groups, and since the pathology of these has a considerable bearing on the treatment I propose to go into them in some detail.

On November 14th, I saw George O., aged 33, a printer. He complained of tightness round the heart, some shortness of breath, much palpitation. These had been worse for six days; he could not say how long they had gone on. He had never had rheumatism or rheumatic fever, and remembered no previous illness. He was a very heavy drinker. On examination a greatly enlarged heart was found, extending below the seventh rib a little outside the nipple line. A well-marked double murmur was audible at the base of the heart, and a suspicion of thrill was palpable. Durozier's sign could be heard in the femorals, and the vessels in the neck showed visible pulsation. The pulse was not definitely of a water-hammer character. No capillary pulse could be detected, nor could the second sound be heard in the vessels of the neck. The case was diagnosed as one of aortic regurgitation with possibly slight stenosis. On November 27th, while undertaking rather violent muscular exertion he suddenly dropped down dead.

What was the cause of this man's sudden death? I have already pointed out the great strain that the blood regurgitating from the aorta during diastole throws upon the dilated left ventricle, and I have drawn your attention to the fact that the work the ventricular muscle has to do varies with the cube of the radius of curvature of the ventricle, so you will easily understand that a point may be reached in any case of aortic regurgitation where a comparatively slight, sudden increase in the dilatation of the ventricle causes the work demanded from the ventricular muscle to be more than it can accomplish. Possibly a feeble abortive contraction takes place; possibly the muscle refuses to respond at all, but the heart stops and the patient falls suddenly dead. In this man's case his heavy drinking had probably increased the degeneration in the cardiac muscle, and the rise of blood-pressure produced by the muscular exertion in which he indulged just before he dropped dead was enough to bring the ventricle to a standstill.

### Lines of Treatment.

I need not detail the post-mortem findings; suffice it to say that he was a man who died as the result of aortic disease, and aortic disease alone; the other valves had not become secondarily affected. You see the immense importance of doing everything possible to avoid an increase of the dilatation of the left ventricle. Of course, we must avoid everything which tends to produce degeneration of the muscle, but it is obvious that in increased resistance to the flow of blood through the arteries and capillaries to the veins lies the greatest danger. Where this is high, the left ventricle is called upon to make a greater effort during systole, and is exposed to a grèater strain during diastole than if it is low. In cases of pure aortic regurgitation, therefore, our efforts should be directed to lowering this resistance, so that the amount of force required during systole to keep up the circulation is as low as possible. My own practice is to give a mixture containing bromides and iodides to these cases. I have never been able to convince myself that iodide of sodium has any practicable superiority over iodide of potassium; either will do, but I am sure that the addition of bromide of potassium is advantageous, possibly partly because it quiets the nervous system, and we know that the blood-pressure is largely influenced by the emotions. The various vaso-dilators, such as amyl nitrite, trinitrin, erythrol tetranitrate are also useful, but should not, I think, be given as a matter of routine. Belladonna combined with bromide and iodide is often beneficial. Where there is sleeplessness, as is not infrequently the case, a hypodermic injection of morphia is often of the greatest service. Digitalis should not be given in this class of case; the great and chief action of digitalis is to increase the tonus of the vascular muscle, and while it is true that it may somewhat help the muscle of the ventricle, on the whole it does more harm than good by increasing the contraction of the peripheral arterioles and possibly also lengthening the diastole. I have more than once seen patients in the stage of aortic disease under discussion relieved of pain down the arm and in the chest by stopping the digitalis which had been injudiciously given. It is said that strophanthus causes less peripheral constriction than digitalis, and so is safer, but even this drug, I think, is better avoided if the mitral valve is competent. The heart muscle may be sustained and stimulated, if desirable, by strychnine, but remember strychnine is incompatible with iodide of potassium, as it forms an insoluble iodide. You can, however, if you wish, prescribe them together if you add a drachm of alcohol to each ounce of the mixture, as the alcohol will keep the iodide of strychnine in solution. I need not dilate on the importance of avoiding sudden and severe muscular exercise, violent emotion, or other like causes of increased blood-pressure, nor the importance of the patient pursuing a quiet, even life, as these will be obvious to you all.

There is, however, another class of case where aortic regurgitation has gone on for some time, producing the conditions in the ventricle already described, wihout any sudden rise of pressure causing sudden death, but where as the result of the gradual dilatation of the left ventricle the mitral valve becomes incompetent; when this occurs, the danger of sudden and dramatic death is to a certain extent lessened, but a new series of symptoms arise. Before, the difficulty which the left auricle had to face was that of pouring, during its systole, the blood it contained into a possibly partly filled ventricle, but now added to this it has a stream of blood under pressure forced into it during its diastole by the contraction of the ventricle, consequently the auricular dilatation increases and the pulmonary blood-pressure rises, so greatly increased work is thrown upon the right ventricle, and changes occur in the right side of the heart. For a time the increased pulmonary pressure may be compensated by hypertrophy of the right ventricle, but in a heart already seriously altered from aortic regurgitation this compensation is not, as a rule, long maintained; the right ventricle dilates, tricuspid regurgitation takes places, and we get engorgement of the systemic venous system with the usual symptoms of enlarged liver, œdema of the legs, and possibly ascites; in fact, the case which was primarily aortic regurgitation now puts on the symptoms of mitral disease and terminates as mitral cases do.

This is the class of aortic case in which digitalis and its congeners are useful. The peripheral vaso-constrictor action of the drug is of less importance, because the regurgitation through the mitral orifice acts to a certain extent as a safety valve. Consequently when the left ventricle contracts there is not the same fear of it being unable to complete its contraction, so there is less risk of a sudden strain bringing it to a standstill, while the beneficial effect of the drug on the cardiac muscle is more marked because the right ventricle is now becoming embarrassed and needs its assistance. Speaking generally, we may say, when in a case of aortic regurgitation the mitral valve has given and the right ventricle is becoming embarrassed, digitalis or strophanthus are beneficial. The signs of failure of the right ventricle are, of course, those of pulmonary engorgement, enlarged liver, and œdema. But while we give digitalis in these cases, there is no reason why we should not at the same time endeavour to lessen the peripheral resistance, and the action of the digitalis should be carefully watched. The large doses permissible and advisable in cases of pure mitral regurgitation should not be given, and directly the right ventricle has somewhat recovered itself the drug should be stopped, for you will easily see that if we have toned up the left ventricle sufficiently to stop, even for a time, the mitral regurgitation, all the dangers of digitalis referred to before again come into play.

